# The first isolation and detection of *Ornithobacterium rhinotracheale* from swollen head syndrome-infected broiler flocks in Iraq

**DOI:** 10.14202/vetworld.2021.2346-2355

**Published:** 2021-09-07

**Authors:** Baraa Akeel Al-Hasan, Abdullah O. Alhatami, Husam Muhsen Abdulwahab, Ghadeer Sabah Bustani, Eman Abdul Wahab Alkuwaity

**Affiliations:** 1 Medical Laboratory Technology Department, College of Medical Technology, The Islamic University, Najaf, Iraq; 2 Department of Microbiology, Faculty of Veterinary Medicine, University of Kufa, Najaf, Iraq; 3 Department of Pathology, Faculty of Veterinary Medicine, University of Kufa, Najaf, Iraq; 4 Department of Physiology and Pharmacology, College of Nursing, Altoosi University College, Najaf, Iraq; 5 Department of Physiology, Biochemistry, and Pharmacology, College of Veterinary Medicine, University of Baghdad, Iraq; 6 Department of Chemistry and Biochemistry, Faculty of Medicine, Jabir Ibn Hayyan Medical University, Najaf, Iraq

**Keywords:** broilers flocks, culture method for isolation and detection *Ornithobacterium rhinotracheale*, Iraq, *Ornithobacterium rhinotracheale*, polymerase chain reaction, reverse transcription-polymerase chain reaction, Swollen head syndrome

## Abstract

**Background and Aim:**

The swollen head syndrome (SHS) makes up complex diseases that infect the upper respiratory tract in poultry and causes several economic losses. Furthermore, this syndrome is considered one of the multifactorial etiological agents. Therefore, this study isolated and molecularly detected *Ornithobacterium rhinotracheale* (ORT) in poultry.

**Materials and Methods:**

This study was conducted at 67 broiler farms that had birds observed to be infected with the SHS from September 2018 until August 2019. Subsequently, swabs were collected from their trachea, infraorbital sinuses, and lungs, after which obtained samples were treated through two methods: (a) The direct method, by uploading samples on FTA cards, and the indirect method using a transport media. Afterward, reverse transcription-polymerase chain reaction (RT-PCR) was used to analyze the directly treated samples; howeverAQ1, the culture method, followed by PCR, was used to analyze the indirectly treated samples. Next, a partial *16S RNA* gene was isolated using four positive PCR products, after which the effect of 16 antibiotics was studied on the seven local ORT strains isolated.

**Results:**

The quantity of ORT isolated using the direct method was 28 (41.7%) samples, which were all positive for the strain. Identification was by direct molecular identification (RT-PCR) from samples loaded on FTA cards. Alternatively, 7 (10.4%) ORTs were detected from the indirect method, as obtained using the culture method and biochemical tests. Then, PCR was subsequently used to confirm the results. As observed, 784 bp bands were shown for all seven ORT isolates. Furthermore, results revealed a significant difference in the detection of ORT strains between direct and indirect methods, with p-value (<0.05) and standard deviation of the error±0.038 for the direct, then ±0.061 for the indirect method. For further analysis on the strain types, four 784 bp PCR products were taken, then partial 16S ribosomal sequence typing was conducted. All these four strains were found to be recorded in NCBI for the 1^st^ time as a local Iraqi strain, with accession numbers (MN931657, MN931656, MN931655, and MN931654). Notably, results also showed that all isolated strains were multidrug-resistant.

**Conclusion:**

From the results, ORT is proposed to be implicated as one of the etiological factors that cause SHSs in poultry. Phylogenetic analysis of the current ORT bacterial strains also showed that they are closely related to the Egyptian isolates.

## Introduction

*Ornithobacterium rhinotracheale* (ORT) is a Gram-negative, pleomorphic, and rod-shaped bacterium causing upper respiratory diseases in the commercial poultry industry. It belongs to the superfamily VrRNA, and it is among the family of the *Flavobacteriaceae* that has its origin from the *Cytophaga lavobacterium*. Reports have highlighted that bacteroides descending genetic line; ORT [1], was isolated for the 1^st^ time in Germany, in the year 1981 from 5-week old turkeys showing nasal discharge, facial edema and fibrinopurulent air sacculitis [2]. Another report also highlighted the first isolation of this strain in the United States in 1989; subsequently, in 1993, it was first characterized. However, in 1994, after isolating and evaluating 21 strains associated with various respiratory tract infections, the ORT was given its name [3].

ORT was previously knoFwn as pasteurella-like, kingella-like, pleomorphic Gram-Negative Rod, or TAXON 28. Nevertheless, after extensive DNA-rRNA hybridization analysis, it was determined that the taxon should be placed on a separate phylogenetic branch within the rRNA superfamily V. Consequently, attention was also given to differentiating ORTs from *Riemerella anatipestifer* and *Capnocytophaga* species because of their similarities in phenotypic characteristics. Hence, although these strains were not closely related, Ornithobacterium, Reimerella, and Coenonia were all grouped as members of the family *Flavobacteriaceae* due to their phylogenetic lineages [4,5]. As a result, 18 known serotypes (A–R) of ORT currently exist as determined by agar gel precipitation serotyping methods [6,7].

Recent studies conducted in neighboring countries, including many provinces of Turkey, have identified ORT. From these studies, ORT was shown to possess different randomly amplified polymorphic DNA and protein profiles [8]. In contrast, the prevalence of ORT in broiler flocks in southwest Iran was (27.39%) [9]. However, according to a cross-sectional study conducted in Jordin, the prevalence was (21%) in commercial broilers [10]. Likewise, in Egypt, the prevalence was (18.6%) in broilers as obtained from the biochemical and serological tests conducted [11]. Nevertheless, no reports of ORT in Iraq before now.

Studies have reported that many Ornithosis features were variable, like the severity of clinical manifestations of the disease in addition to duration or mortality rates. However, these manifestations depended on many factors, such as live viral infection burdens or other bacterial synergism occurring during the infection process. Furthermore, many important factors, such as stock density, bad management, and environmental conditions accounted for infection rates, as the ORT infection was also reported after stress factors like live vaccination [7,12]. Nevertheless, the main characterized symptom of swollen head syndrome (SHS) was observed to be swelling of the head and facial edema, which resulted from accumulating inflammatory exudates beneath the skin of the head [13]. Further manifestations reported included difficult breathing, coughing, rales, swollen infraorbital and supraorbital sinuses, including conjunctivitis and severe depression. In addition, the main gross lesions demonstrated among infected chickens, included gaseous exudates in the trachea, nasal passages, and sinuses. Yellowish gaseous exudate on the air sacs, ovaries, and the peritoneum has been reported as well, with mortality rates varying depending on secondary etiological infections [14].

On the basis of these symptoms, it was important to properly identify the ORT strains. Therefore, for isolating and detecting ORTs, many previous studies had recommended the use of blood agar with 10 mg/mL gentamicin in the presence of 5-10 CO_2._ Pinpoint grayish-white small colonies indicated the possible presence of ORT [7]. Biochemical tests conducted also revealed that ORT isolates were catalase-negative and oxidase-positive. This observation was confirmed by culturing the suspected colonies on MacConkey’s agar medium, which revealed negative results in the presence of ORT [15]. Nevertheless, using the polymerase chain reaction (PCR) diagnostic method has shown to be one of the most important detection methods for ORT due to its proven sensitivity and accuracy compared to traditional isolation methods [12,15-17]. Likewise, for further confirmation of the presence of ORT, *16S rRNA* sequencing has been conducted in several studies. This aspect has also been relied upon for diagnosis due to the *16 S rRNA* gene present in all bacteria. The *16S rRNA* gene comprises highly conserved nucleotide sequences, interspersed with variable regions that are genus- or species-specific. PCR primers targeting the conserved regions of these rRNAs can then amplify the variable sequences of the *rRNA* gene [18]. In contrast, reverse transcription PCR (RT-PCR) is among the reliable diagnostic methods for ORT that has allowed to determine the quantity of etiological agents [19].

Thus, during this study, two methods of isolation and identification of bacteria were followed. The direct method using RT-PCR after DNA extraction from the samples directly, and the indirect method, conducted by following the previously mentioned steps (culturing bacteria, then confirmation using biochemical tests and PCR). We designed this research to isolate and molecularly detect ORT with these objectives:

· Investigating the prevalence of ORT in broiler flocks that were infected with SHS.
· Partial 16S ribosomal sequencing for the ORT strain present in our country to detect the matching degree of this local strain with those from neighboring countries.
· Comparative analysis between the direct and indirect methods for ORT detection.

## Materials and Methods

### Ethical approval

The experiments of the present study were reviewed and approved by Institutional Animal Care and Use Committee, Faculty of Veterinary Medicine, University of Kufa, Iraq (8689-2020).

### Study period, area, and sample collection

The collection of samples was conducted beginning from September 2018 till the end of August 2019 from 67 broiler farms. The field of the study surveyed was the middle Euphrates region (Baghdad 2, Wasit 10, Karbala 14, Al Muthanna 7, Al-Najaf 13, Al-Qadisiyyah 21). Included broilers aged between 3 and 6 weeks old. Then, three to four SHS cases were taken from each farm and pooled together as one sample. Subsequently, samples were collected from their lungs, air sacs, and infraorbital. These obtained samples were simultaneously preserved on a transport media and uploaded on FTA cards.

Next, samples were divided into two groups. The first set was those put in a transport media (indirect method), which was transported to thelaboratory at the Faculty of Veterinary, University of Kufa, for culturing and detection, and the second group comprised those samples put on FTA cards (direct method) to preserve the genetic materials therein for microorganism detection using RT-PCR.

### Medias for bacterial suspension preparation and re-isolation

All media (solid and broth) were prepared by sterilization through autoclaving according to the manufacturer’s instructions (HiMedia, India). To isolate the ORT, samples were cultured primarily using a nutrient broth, after which the tubes were placed inside an anaerobic jar at 37°C for 24-48 h [3,20,21]. Subsequently, specimens were then cultured on a blood agar base plate and supplemented with 10% sheep blood agar. To the setup, 10 mg of gentamycin/mL of blood agar was also added to avoid bacterial contamination, and then the culture was incubated at 37°C for 48 h under anaerobic or microaerophilic conditions [22,23].

Afterward, the suspected colonies were cultured on MacConkey’s agar medium under similar environmental conditions. After 48 h, positive ORT cultures will be unable to grow on the plates [15,24,25].

### Biochemical tests

The urea slant was stabbed, and streaks were made using the bacterial colony. For urease tests, the tubes were incubated for 24-48 h. at 37°C. A color change in the medium to pink indicated positive results [26]. Likewise, oxidase test swabs from hardy diagnostics were used for the oxidase test. However, for the catalase test, 3% H_2_O_2_ was used [27]. The previous studies were followed and conducted for the other biochemical tests conducted in this study [8,24].

### Antibiotic sensitivity

A group of antibiotic disks having different concentrations was used for this analysis, following the protocol by Bioanalyse, Turkey as shown in Table-1. The choice of the antibiotic disk to be used was considered more than their availability in Iraq during the selection of antibiotics, then, the most common among veterinarians were obtained.

**Table 1:** Antibiotics and their antibiotic content.

Antibiotic	Symbol	Content (mcg)
Amoxicillin	AX	25
Ampicillin	AM	25
Azithromycin	AZM	15
Chloramphenicol	C	30
Ciprofloxacin	CIP	10
Colistin	CT	10
Doxycycline	DO	10
Enrofloxacin	ENR	5
Erythromycin	E	10
Gentamicin	CN	10
Levofloxacin	LEV	5
Oxacillin	OX	5
Tilmicosin	TIL	15
Trimethoprim	SXT	1.25
Sulfamethoxazole		23.75
Tylosin	TY	15
Vancomycin	VA	30

### Molecular characterization

ORT DNA was extracted from nucleated cells under aseptic conditions following the protocol of the Favorprep™ DNA Extraction Kit, Korea. Similar recommended steps were also followed to extract DNA from FTA cards. Subsequently, to detect ORT in the samples, PCR was used. The protocol used for the PCR analysis depends on the abm^®^ manufacturer’s instructions. In this study, our final reaction volume was 50 mL. Designing of primer sequence and PCR conditions used were also conducted following the method reported in Hafez’s study [5,28,29].

Next, to detect ORT, real-time PCR (RT-PCR) was done. It was prepared according to the manufacturer’s instructions (Gentix, Germany), where specific DNA sequences from the ORT genome are amplified, then the generated PCR-product was detected using an oligonucleotide probe labeled with fluorescent dyes [23,30].

### Statistical analysis

#### Statistical data analysis and presentation

Data were analyzed and presented using PRISM, GraphPad 8, Numbers application for MAC 11, Statistical Package for the Social Sciences 16.0 (IBM Corp., NY, USA), and Microsoft Excel 2010. Subsequently, the obtained data were checked for normal distribution using Shapiro–Wilk’s test. A mixed-model analysis of variance (t-test and one-way analysis of variance [ANOVA]) was then used to compare the differences between means among the variable groups, after which significance was tested using the same mixed model (t-test and one-way ANOVA). Values less than 0.05 were considered statistically significant. Data were presented as mean±­standard deviation of the error.

#### Evolutionary relationships between BAH species of the local strain

The evolutionary history was inferred using the neighbor-joining method [31]. Furthermore, the bootstrap consensus tree was inferred from 500 replicates [32], which was then taken to represent the evolutionary history of the taxa analyzed [33]. Subsequently, branches corresponding to the partitions that were reproduced in less than 50% bootstrap replicates were collapsed. Then, the evolutionary distances were computed using Jukes–Cantor’s method [34]. Units of the number of base substitutions per site were used. In this study, the analysis involved 106 nucleotide sequences, where the codon positions included were 1^st^ + 2^nd^ + 3^rd^ + Noncoding. In addition, all positions containing gaps and missing data were eliminated. Finally, 686 positions in the final dataset were obtained. Evolutionary analyses were then conducted using MEGA7 [35].

## Results and Discussion

### Prevalence of ORT strains from obtained samples in middle Euphrates region

The direct method was used to detect ORT strains in the middle Euphrates region. As highlighted previously, the samples had previously been uploaded on FTA cards after DNA extraction, then detected using RT-PCR. Results showed that ORT were detected in 28 (41.7%) samples, whereas, 39 (58.2%) samples were negative for ORT.

Further investigations revealed that the 28 (41.7%) positive ORT strains were distributed within five governorates, including Al-Najaf 8 (28.57%), Wasit 10 (35.71%), Karbala 3 (10.71%), Al-Muthanna 1 (3.57%), Al-Qadisiyyah 6 (21.42%), and Baghdad (0).

This result was expected in Iraq due to the recorded cases of infection in the neighboring Iraq countries [7,10,15,16,36,37]. Therefore, we propose that its isolation for the 1^st^ time in the middle Euphrates region was predictable as a result of the establishments of broiler farms and the high consumption for broiler meat, which led to a high abundance in commercial exchange between the provinces of the middle Euphrates. We also propose that the import of broiler meat products from neighboring countries accounted for the observed prevalence. In addition, we propose as well that among the reasons that helped the spread of ORT in Iraq was the absence of slaughterhouses dedicated to poultry. Furthermore, a large percentage of consumers refrained from eating frozen poultry products and preferred to buy live broilers and slaughter them at home. It has also been observed that importing chicken eggs from these countries accounted for the observed prevalence. Hence, we conclude that what strengthened these causes was the vertically and horizontally rate of bacterial transmission as proposed by a previous study as well. In agreement, their study showed that ORT was transmitted by aerosol and egg [5].

### Detection of ORT in using the direct method

The method for detecting ORT strains in this study was conducted using RT-PCR directly from FTA cards, after which DNA extraction was done. Then, the results obtained were confirmed using BioDrop spectrophotometer (UK). Subsequently, the samples were identified using RT-PCR. After completion of the programmed cycles, ORT results were shown using a FAM-TAMRA dye. Primers used for ORT detection and are shown in Table-2.

**Table 2:** The results of cycle threshold value for samples+ORT.

Number	DNA for samples	CT for ORT positive
1	W6	31.2
2	W7	35.0
3	Q17, Q18, Q19	35.2
4	N3	32.9
5	N4	33.0
6	Q2	28.5
7	Q3	32.9
8	M3	33.5

CT=Cycle threshold, ORT=*Ornithobacterium rhinotracheale*

**Figure-1:**
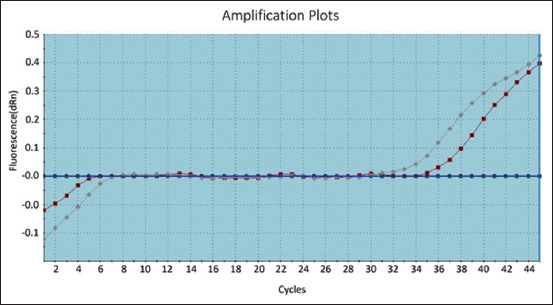
Amplification curves for positive W6 and W7 on reverse transcription-polymerase chain reaction.

**Figure-2:**
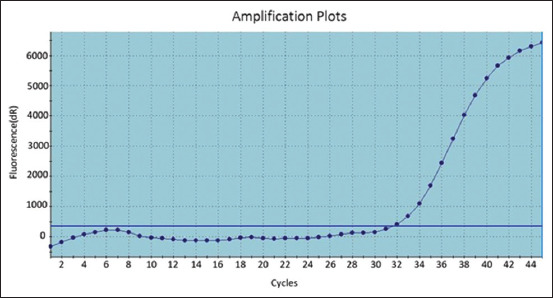
Amplification curves for positive M3 on reverse transcription-polymerase chain reaction.

**Figure-3:**
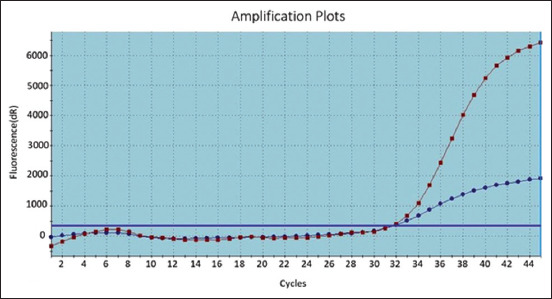
Amplification curves for positive N3 and N4 on reverse transcription-polymerase chain reaction.

**Figure-4:**
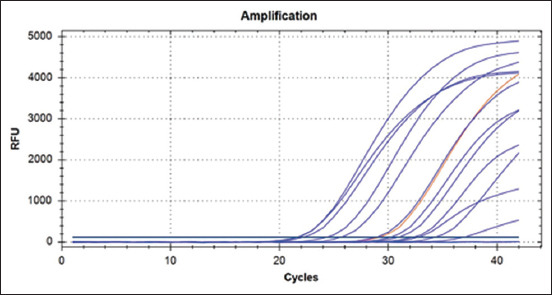
Amplifications curves for positive Anicon results for *Ornithobacterium rhinotracheale*.

As shown in Figures-1-3, the positive amplification curves of some of the positive ORT in isolates were detected using RT-PCR. Subsequently, groups of FTA cards were sent to the ancon laboratory in Germany to confirm the isolation of ORT strains. Results are shown in Table-3 and Figure-4.

**Table 3:** Ancon laboratory cycle threshold value for samples+ORT.

Number	Samples	CT for ORT positive
1	W1, W2	32.7
2	W3	34.3
3	W4	31.0
4	W5	21.6
5	W6	22.2
6	W7	28.9
7	W8	24.3
8	W9, 10	25.6
9	K7, K8, K9	21.7
10	N1, N2, N3, N4	37.0
11	N5, N6, N7, N8	32.2
12	Q1, Q2, Q3	30.2

CT=Cycle threshold, ORT=*Ornithobacterium rhinotracheale*

This study mainly isolated and identified ORT strains present in Iraq. Building on previous studies, it had been proven that isolating bacteria on a culture media was difficult and inaccurate due to many causes, which was also founded during this study. These issues will be mentioned later [1]. Therefore, we resorted to the direct method of detection using RT-PCR. This method was selected because of its higher specificity and sensitivity [19]. Furthermore, this method was adopted to ensure that the DNA was not lost while handling the sample. In this way, the acquisition of bacteria to be diagnosed was large. The previous studies also agreed with us based on their results [38]. We also found that a cycle threshold value of diffraction existed between the samples due to the differentiation of ORT’s DNA in the sample [39]. We propose that this was due to the fact that some of the FTA cards well were used to upload more than one positive ORT. Thus, during DNA extraction we observed that the constriction of ORT’s DNA was high, and the cycle threshold values were less than the others.

### Detection of ORT using the indirect method

From our study, using the indirect method (transporting the media, then culturing and PCR) isolated positive specimens of the ORT strain. Seven of the isolates were ORT positive, whereas, the 60 other bacterial strains were negative. These results were obtained after culturing on different types of media, after which we detected the isolated therein using biochemical tests and PCR. Further investigations showed that two each of the specimens originated from Al-Najaf, Al-Qadisiyyah, and Wasit, whereas, one was from Al-Muthanna. Significant differences were also found between the direct and indirect methods. p-value (<0.05), and standard deviation of the error±0.038 was obtained for the direct method, then ±0.061 was obtained for the indirect.

Based on our results, we propose that the reason for the decrease in bacterial isolates was due to the fact that ORTs were difficult to isolate on a culture media [15] because it required 48-72 h to grow on a media. This difficulty was also dependent on its ability to form small colony variations [27]. Furthermore, due to the rapid and heavy growth of other bacteria colonies, which led to their coverage ORT, in addition to the small size of the ORT colonies compared to other bacterial strains, difficulties have been observed [40]. Due to these causes, an increased rate in the complexity of isolating ORTs was observed [16,41], which was also dependent on the high resistance of bacteria in samples similar to gentamicin at a concentration of 10 mg/mL. These observed features served as an obstacle to ORT isolation. Alternatively, depending on a previous study SHS in poultry, possessed pathogenic bacteria having the property of multiple drugs resistance. However, a conducted study found that *Escherichia coli* strains isolated from an SHS outbreak possessed multiple drugs resistance [14]. Therefore, we propose that what made isolation and identification of ORT more difficult was the inability to inhibit other bacterial growths [16].

### Biochemical tests for ORT

Results from the biochemical tests it was decisive according to 22 suspected ORT strains that did not grow on the MacConkey agar. However, we observed that the outcome from the seven positive isolates shared similar outcomes in biochemical test results, as shown in Table-4.

**Table 4:** Biochemical test results for *Ornithobacterium* *rhinotracheale* isolates.

Test	Result
Grow on blood agar	+
Growth on MacConkey agar	-
Hemolysis	-
Gram staining	Negative
Catalase	-
Oxidase	+
Indole production	-
Urease	-
Motility	-

Results obtained through biochemical tests also indicated a high level of agreement with those obtained from most researchers [7], as most of the ORT isolations grew on blood agar as the main agar [16]. However, some studies demonstrated the susceptibility of bacteria to blood analysis, this feature had not been attributed with ORT. This advantage has been observed to be due to an iron acquisition mechanism with which the pathogen overcomes a host’s ability to limit iron during infections [42]. Furthermore, the results from previous studies led to the hemolysis of blood agar accrued after 48 h [27]. However, our study did not wait until that time.

For the other biochemical results, many studies agreed with our results [43]. Nevertheless, the ORT catalase test was negative. We propose that this result was due to the lack of producing the catalase enzyme [26]. Alternatively, the seven other isolates were oxidase-positive, which was also linked to the availability of cytochrome c oxidases [27]. Other biochemical tests were identical to those from the previous study [11]. In addition, we propose as well that the reason for each negative result for indole and urease tests were both due to the lack of ORTs to produce tryptophanase, which catalyzes the hydrolyzes of urea to produce ammonia and carbon dioxide [26].

### Detection of ORT by PCR

**Figure-5:**
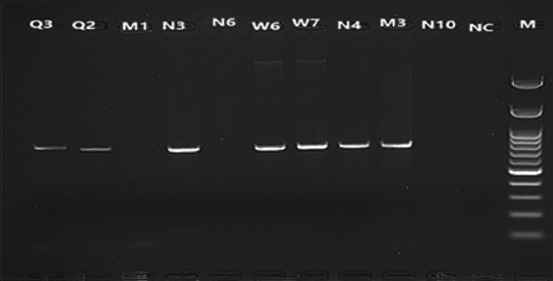
Ethidium bromide-stained agaros gel of polymerase chain reaction results for *Ornithobacterium rhinotracheale* 16S ribosomal RNA, using DNA extracted by Favorprep™ DNA Extraction Kit, lanes coded according to strain designation code. Lane M 100 bp plus Opti-DNA Marker. Lane NC: No template control negative, A: Lanes (Q3,Q2,N3,W6,W7,N4,M3) positive for *Ornithobacterium rhinotracheale*, B: lanes (M1, N6, N10) negative for *Ornithobacterium rhinotracheale*.

The biochemical tests results were confirmed using PCR. Primers adopted here depended on those reported by a previous study [44]. All ORT strains suspected to be isolated were positive, as 784 pb amplification products were obtained, which corresponded to the expected size. Results also showed that the number of amplified products was seven obtained from the negative control and samples using a 100 bp marker. Results are shown in Figure-5.

This study mainly isolated the ORT as a respiratory pathogen in poultry. Therefore, we used PCR to ensure that isolation and results obtained conformed with those of several studies having similar PCR products (784 bp) [1].

### Sequence typing of ORT

For further confirmation of the four samples that were positive for PCR, 784 pb were taken. Then, these molecules were subjected to partial 16S ribosomal sequence typing. Subsequently, we compared our results with 100 other ORT results obtained from the National Center for Biotechnology Information**.** Results were recorded, after which the four new ORT strains obtained were confirmed for the 1^st^ time in Iraq, as shown in Table-5.

**Table 5:** The sequence of the local isolate strain of *Ornithobacterium rhinotracheale* that was registered in NCBI.

Number	Sequence code	Governorate	Collection date	Accession number
1	BAH_M3	Al-muthanna	17-Nov-2018	MN931654
2	BAH_N3	Al-najaf	20-Oct-2018	MN931655
3	BAH_N4	Al-najaf	20-Oct-2018	MN931656
4	BAH_W7	Wasit	20-Apr-2019	MN931657

The strain BAH_W7 showed 99.87% identity with ORT strain from Egy 1 16S ribosomal RNA, with a score of 1375. In addition, the accession number was MG773129.1. Comparing this strain with those of previously isolated ones (BAH_M3, BAH_N3, BAH_N4), their identity were similar as well (Table-6).

**Table 6:** Isolated strain and their identic strain with percentage of identity.

Strain code	Percentage of identity	Identic strain	Total score	Accession number	Query cover (%)	Country
BAH_M3	99.58%	DW-2	1297	MN023015	98	China
BAH_N3	99.71	DW-2	1275	MN023015.1	100	China
BAH_N4	99.58	DW-2	1303	MN023015.1	100	China
BAH_W7	99.87	Egy 1	1375	MG773129.1	100	Egypt

The partial 16S ribosomal sequence typing was essential in our study. This verification technique has also been supported by many studies that use partial 16S ribosomal sequence typing methods to identify the source of isolation [40]. In addition, we can also determine the percentage of mutations in the local strain through comparison with other strains in the National Center for Biotechnology Information gene bank [45].

Our results agreed with [3], where he showed in his study that a percentage identity of 99.87% was approximately identical. Furthermore, the remaining samples followed with a different match rate, and the reason for this was due to the susceptibility of bacterial strains to create genetic mutations that are proposed to lead to a slight change in the genes [7].

**Figure-6:**
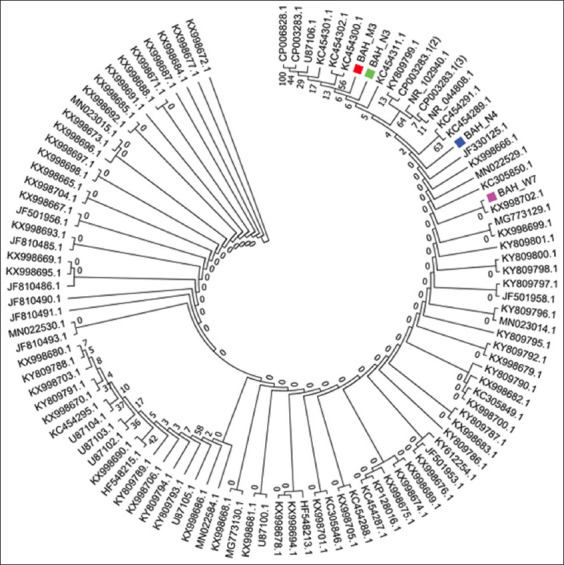
Local strain of *Ornithobacterium rhinotracheale* and their relationship with other strains.

**Figure-7:**
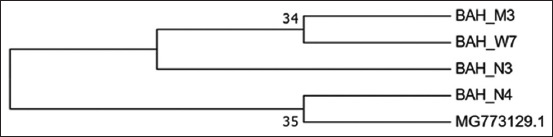
Genetic relationship between Egyptian stain and local strain.

**Figure-8:**
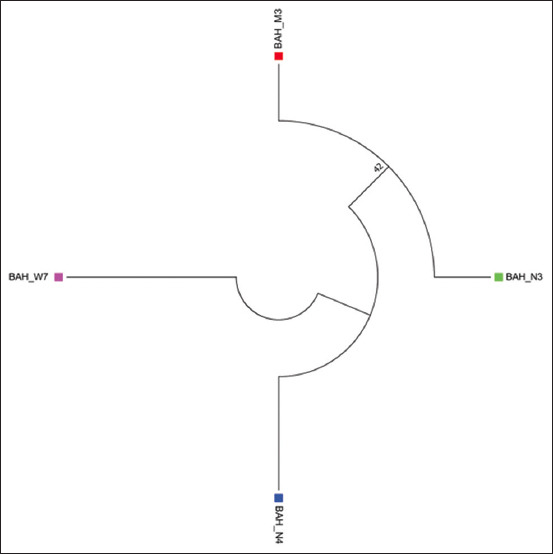
Relationship between all local strain.

The relationship that exists has also been founded between the strains as depicted on the genetic tree for ORT shown in Figure-6. As shown (Figure-6), the local strain (BAH_W7), had the highest percentage identity with the Egyptian strain (Egy 1), with accession number (MG773129.1). Furthermore, as shown in Figures-7 and 8, the most identical local strains were BAH-N4 to BAH-W7, which started to spread in Al-Najaf, thereby forming the BAH-N3 strain with some gene mutation. Subsequently, the local strain (BAH-N4) mutated other strains located in Al-Muthanna, as shown in Figure-8. All these ORT distributions in differing governorates were due to the transition of broilers between the different areas without control. Furthermore, screening tests exist for ORTs in Iraq.

### Effect of antibiotics on ORT

The seven positive PCR strains obtained were cultured on a Mueller Hinton Agar using 16 antibiotics to study their antibiotic susceptibility for local ORT strains. Results showed that all seven local strains were 100% resistant to (azithromycin, colstin, erythromycin, gentamicin, oxacillin, tilmicosin, tylosin, and vancomycin). Hence, we classified the ORT as multidrug-resistant bacteria [39]. In contrast, all other local strains were 85.7% resistant to (amoxicillin, ampicillin, chloramphenicol, and trimethoprim-sulfamethoxazole). Other studies reported similar results [46].

Our results also showed that the isolates had a high resistance rate (71.4%) for each of (doxycycline and ciprofloxacin), which was in agreement with the results obtained from a study conducted on laying hens in India [47]. Furthermore, 28.5% of the currently isolated strains of ORT were resistant to enrofloxacin. However, results from our study disagreed with those obtained by a study [39] because all their strains were resistant to enrofloxacin. Notably, in our study, levofloxacin was the drug of choice due to the fact that all local strains were unresistant to this antibiotic as shown in Table-7.

**Table 7:** Resistant percent of each ORT to each antimicrobial agent used in this study.

Antibiotics	Percentage of antibiotic resistant (%)	Samples*						

		Q3	Q2	N3	W6	W7	N4	M3
Azithromycin	100	R	R	R	R	R	R	R
Colistin	100	R	R	R	R	R	R	R
Erythromycin	100	R	R	R	R	R	R	R
Gentamicin	100	R	R	R	R	R	R	R
Oxacillin	100	R	R	R	R	R	R	R
Tilmicosin	100	R	R	R	R	R	R	R
Tylosin	100	R	R	R	R	R	R	R
Amoxicillin	85.7	R	R	R	R	R	R	S
Ampicillin	85.7	R	R	R	R	R	R	I
Chloramphenicol	85.7	R	R	R	R	R	R	I
Doxycycline	71.4	R	I	R	R	I	R	R
Enrofloxacin	28.5	I	S	S	S	R	I	R
Trimethoprim sulfamethoxazole	85.7	R	R	R	R	R	R	I
Vancomycin	100	R	R	R	R	R	R	R
Levofloxacin	0	S	S	S	S	S	S	S
Ciprofloxacin	71.4	R	I	R	R	R	R	I
Percentage of ORT resistant (%)		87.5	75	87.5	87.5	87.5	87.5	62.5

* Local ORT strains. ORT=*Ornithobacterium rhinotracheale*, S=Susceptible, I=Intermediate, R=Resistant

The reason for levofloxacin’s activity was because levofloxacin was in the class of a later generation of fluoroquinolones, which was collectively referred to as “respiratory quinolones.” This activity distinguished them from the earlier fluoroquinolones, which exhibited modest activities toward important respiratory pathogens. However, the ORT strain has not been found resistant to this yet [48].

It has been reported as well that resistance to ORT antibiotics is varied according to the region from which it was isolated. Thus, the degree of resistance would also differ [37]. However, in France and Belgium, 98% and 71% of the isolates were sensitive to quinolone *in vitro*, respectively [49]. In contrast, ORTs were resistant to quinolone, tetracycline, lincosamide, and macrolide [49]. This result is among the important factors affecting ORT treatments, due to the common use of these antibiotics in Iraq as the first choice for treatment [50,51], given that lincomycin, oxytetracycline, and enrofloxacin had good inhibition rates**.**

According to Tables-3-6, antibiotic resistance differed between the isolates. As shown, local isolates Q3, N3, W6, W7, and N4 were more resistant to antibiotics than others with a percentage of 87.5%, Q2 with a percentage of 75%. In contrast, M3 was less resistant to antibiotics than other isolates, with a percentage of 62. Furthermore, on the basis of other studies, a wide difference has been noted between the results we obtained and what was found from previous studies, where the percentage of bacteria resistance to antibiotics varied. This result was expected due to misuse of antibiotics in Iraq [50], as most owners of broilers used antibiotics randomly, and are proposed to be administering high dosages to healthy flocks as prophylaxis. All these causes would reflected strikingly on bacterial resistance [52,53]. On the basis of several reports, ORT was among many bacterial strains that resisted several generations and families of antibiotics. Nevertheless, many studies agreed that ORT was resistant to gentamicin and neomycin [46,54]. In contrast, our results disagreed with that of a previous study [54], except for M3, which was intermediate to trimethoprim-sulfamethoxazole. However, depending on results from previous studies, our result was beta-lactam resistant because ORTs had several beta-lactamases and possessed multidrug resistance efflux pumps [55]. Comparing these results with that of a previous study, amoxicillin and ampicillin were susceptible to ORT. This finding was, however the opposite of what we found in our study, except for M3 that was susceptible to amoxicillin [56]. Still, our study supported previous results with doxycycline, which had intermediate activities against ORT [57,58].

## Conclusion

Conclusively, ORT is proposed to be implicated as one of the etiological factors that cause SHS in poultry. Furthermore, the phylogenetic analysis of current ORT bacterial strains showed that these strains were closely related to those of the Egyptian isolates.

## Authors’ Contributions

AOA and HMA: Conceptualized, drafted, and supervised the final version as well as editing of the manuscript. BAA: Collected relevant literature, contributed to the original draft, data curation, investigation, and review of the manuscript. GSB and EAWA: Review and editing of the manuscript. All authors read and approved the final manuscript.
